# The effect of temperature on the coupled slow and fast dynamics of an electrochemical oscillator

**DOI:** 10.1038/srep24553

**Published:** 2016-04-15

**Authors:** Alana A. Zülke, Hamilton Varela

**Affiliations:** 1 Institute of Chemistry of São Carlos, University of São Paulo, POBox 780, 13560-970, São Carlos, SP, Brazil

## Abstract

The coupling among disparate time-scales is ubiquitous in many chemical and biological systems. We have recently investigated the effect of fast and, long-term, slow dynamics in surface processes underlying some electrocatalytic reactions. Herein we report on the effect of temperature on the coupled slow and fast dynamics of a model system, namely the electro-oxidation of formic acid on platinum studied at five temperatures between 5 and 45 °C. The main result was a turning point found at 25 °C, which clearly defines two regions for the temperature dependency on the overall kinetics. In addition, the long-term evolution allowed us to compare reaction steps related to fast and slow evolutions. Results were discussed in terms of the key role of *PtO* species, which chemically couple slow and fast dynamics. In summary we were able to: (a) identify the competition between two reaction steps as responsible for the occurrence of two temperature domains; (b) compare the relative activation energies of these two steps; and (c) suggest the role of a given reaction step on the period-increasing set of reactions involved in the oscillatory dynamics. The introduced methodology could be applied to other systems to uncover the temperature dependence of complex chemical networks.

Systems with sub-processes evolving on different time scales are ubiquitous in most natural and man-made systems, such as chemical reactions, neuro-biological and ecological systems, to mention just a few. Although there is a clear distance between these many examples of multiple time-scale systems, they find similarities at the mathematical description level^1^, being often discussed in terms of linearized models, built without necessarily accounting its peculiarities regarding, for example, experimental conditions or physical-chemical nature^2^. In electrochemistry, oscillating surface reactions represent a category of multiple time-scale systems which are fairly useful to investigate complex behavior at its fundamental level^3^,^4^ and in which experimental constraints impact the evolution of temporal patterns. The intricate nonlinear interaction of reactions with disparate time-scales resulting in complex behavior at the solid/liquid electrified interface has been found to be also useful in shedding light on the mechanisms underlying these surface reactions. In this direction, we have recently tackled some intriguing aspects of the complex dynamics of oscillatory electro-oxidation of small organic molecules on platinum and platinum based surfaces^5^,^6^,^7^,^8^,^9^,^10^.

We have recently explored^8^,^9^ the effect of the slow and uncontrollable surface changes in the time-series registered during the electro-oxidation of small organic molecules on platinum. The resulting surface deactivation, similarly to an aging process, is revealed by a gradual and slow drift to more positive values of the electrode potential, acting as a bifurcation parameter that ultimately causes the death of oscillations. We proposed a model for the global oscillatory response in which coupled to the (intrinsic) core oscillator responsible for the oscillations itself, is a rather slow and irreversible process, which is responsible for the abovementioned drift. To compensate this drift, we have proposed an empirical experimental strategy consisting in applying a negative galvanodynamic sweep in order to stabilize the potential oscillations by slowing down the natural drift, increasing the time window for the observable oscillations^9^. Results reported in refs 5,8 and 9 strongly support the physical-chemical picture of the slow accumulation of sub-surface oxygenated species as responsible for the observed drift.

Particularly for the formic acid reaction on platinum, the best model system for investigating fundamental aspects of electrocatalysis of small organic molecules, the interest lies in its relevance for energy conversion systems. The complete oxidation to CO_2_ yields two electrons and only C-H and O-H bonds cleavages are required. However, despite being the simplest reaction among its class and all the extensive amount of available literature on the matter since the classical works from Parsons *et al.*^11^,^12^, mechanistic issues still trigger some heated discussions on the scientific community^13^,^14^. Also, the oscillatory oxidation of formic acid has undergone a long history of experimental investigations and numerical simulations^15^,^16^,^17^,^18^,^19^,^20^,^21^. An intriguing aspect of the oscillatory electro-oxidation of formic acid is the occurrence of temperature (over) compensation^7^. The term was used to define the counter intuitive observation of either a temperature independent relation with the oscillatory frequency or even lower frequencies as the temperature is increased for a certain range of operational conditions.

The present contribution aims to add to the comprehension of the coupled time-scales underlying electrochemical oscillators in terms of the dependence on the bulk temperature. We use the electro-oxidation of formic acid on polycrystalline platinum in acidic media as a model system.

## Results

**Figure 1:**
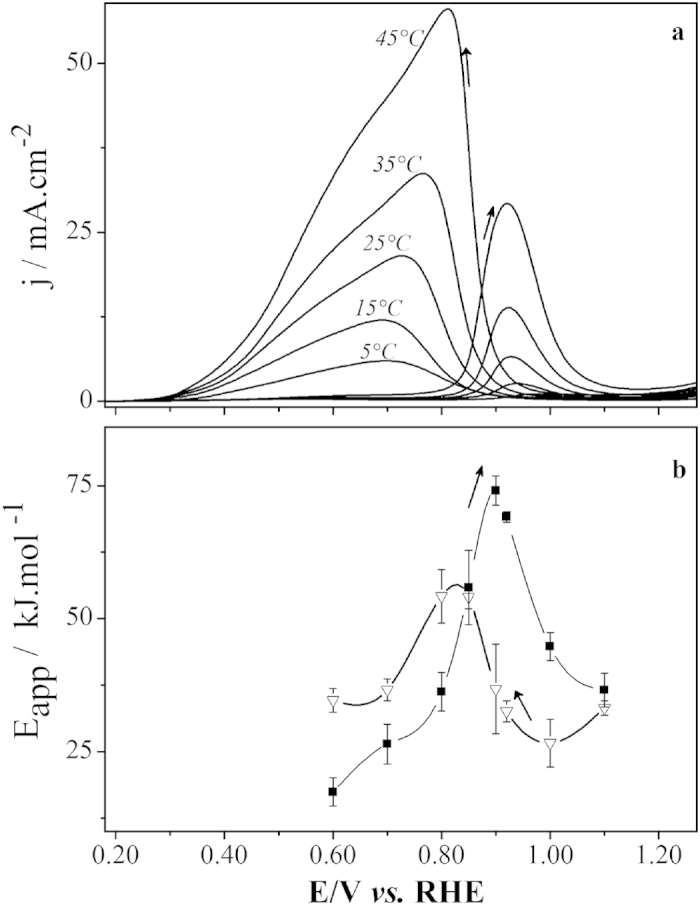
(**a**) Voltammetric profiles (dE/dt = 50 mV.s^−1^) of the system Ptpoly|0.5 M H_2_SO_4_ + 1 M HCOOH at different temperatures as indicated. (**b**) Measured apparent activation energies for the electro oxidation of formic acid in the positive sweep (full squares) and in the negative sweep (empty triangles).

The typical responses upon variation of the bulk temperature on the cyclic voltammetry experiments at 50 mVs^−1^ are depicted in Fig. 1a. Overall, the voltammetric profiles are in agreement with the well-known behavior for the system Pt|HCOOH in sulfuric acid media, showing an oxidation wave with an onset potential of ≈800 mV in the forward scan and a reactivation peak in the reverse scan, starting at about 950 mV^11^,^12^. The contribution of the temperature increase in Fig. 1a is basically seen as an increase in the magnitude of current densities in the whole window. Regarding the hysteresis between positive and negative sweep, no significant effect is observed but a slight decrease of the distance between the direct and reverse peaks follows the temperature increase.

Apparent activation energies (E_app_) were calculated from the slopes obtained by the Arrhenius plots, c.f. Fig. 1b. We estimated E_app_ for the two main oxidative waves as 59 ± 1 kJ.mol^−1^ and 41 ± 2 kJ.mol^−1^, in the positive scan and in the forward scan respectively, showing good agreement with data available in the literature^22^,^23^,^24^,^25^.

**Figure 2:**
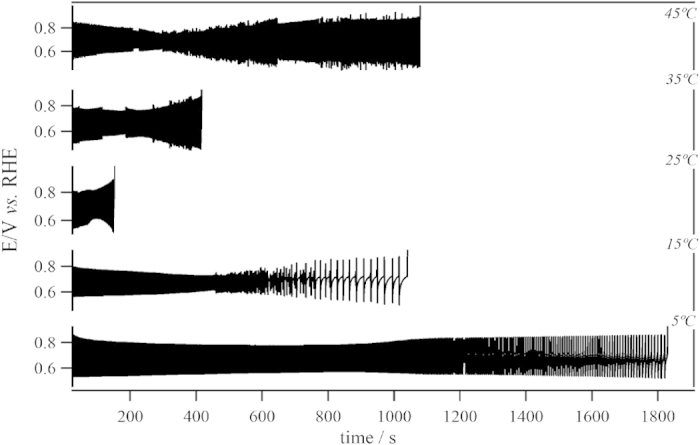
Galvanostatic time-series for a normalized current of 0.5 (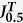, see text for details) and at different temperatures.

Galvanodynamic sweeps at 5 μA.s^−1^ were also performed for five temperatures from 5 to 45 °C, maintaining all other parameters identical. In this case, the obtained time-series were used for mapping the current region in which oscillatory instabilities emerge, allowing us to choose normalized current values 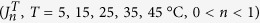 for further galvanostatic investigation, using the procedure suggested by Nagao *et al.*^7^. This normalization procedure allows us to compare a set of potential time-series obtained at different temperatures for a given applied current. Complete time-series of the galvanostatic measurements obtained at different temperatures are displayed in Fig. 2.

**Figure 3:**
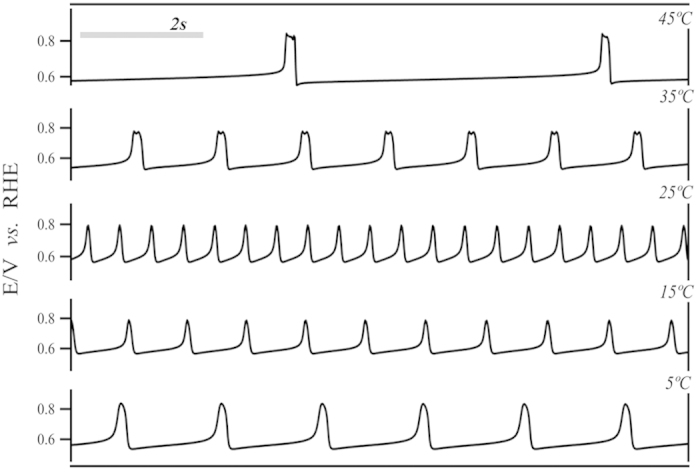
Period length (family F_1_) for different temperatures during galvanostatic time-series (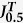).

Typical relaxation-type oscillations with amplitudes ranging from 550 to 850 mV were found for all temperatures^26^,^27^,^28^. Not surprisingly, temperature revealed to be an important parameter in the definition of oscillatory frequency, *f*, potential amplitude, Δ*E* and overall size or stability of the time-series, *S*_*osc*_. For a more reasonable comparison, the complete time-series were divided into three distinguishable families of oscillations (F_1_, F_2_, and F_3_, c.f. Supplementary Fig. S1) that describe the spontaneous evolution of temporal patterns, common to all temperatures investigated here. Potential oscillations starting with high amplitudes of about 300 mV describe well the F_1_ region, which gradually evolve to a region where cycles show lower amplitudes and higher frequencies (F_2_). The transition from F_2_ to F_3_ is marked by an increase in amplitude (Δ*E*) and, again, a decrease in frequency. In F_3_, kinetic instabilities of richer complexity such as mixed-mode oscillations (MMO) were found^27^,^29^. Transition between oscillatory patterns, for instance, from single-period (P^1^) to double-period (P^2^) and periodic-chaotic sequences^2^, were also observed. Those complications led us to opt for oscillatory families F_1_ and F_2_ for further discussions regarding activation parameters, once the well-behaved dynamics presented a better reproducibility for a wider range of experiments. In Fig. 3 is zoomed a representative region for F_1_ highlighting the temperature dependency on the oscillatory dynamics. The overall size of the oscillatory region, *S*_*osc*_, and the oscillatory frequency, *f*, for all three families depend strongly on the bulk temperature. The average oscillatory frequency (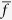) for F_1_ and F_2_ shows the same tendency as *S*_*osc*_: increasing values from 5 to 25 °C with a subsequent decrease from 25 to 45 °C. We have calculated the distribution of frequency by FFT (Fast Fourier Transform) and confirmed that the dominant frequencies display higher values as temperature is increased from 5 to 25 °C, and lower values as temperature is increased from 25 to 45 °C. A more detailed description of the main qualitative and quantitative information extracted from the potential time-series is presented as Supplementary Material (see Supplementary Table S1).

The effect of temperature was also investigated by means of electrochemical impedance spectroscopy (EIS). Plotting the real impedance against the imaginary one, as a function of the modulation frequency *ω*, one obtains an impedance spectrum, which can be used to evaluate the stability of the system. Impedance spectra applying DC potentials from 500 to 1200 mV showed the typical qualitative features known for this system^30^. The negative differential resistance (NDR) region was found between 600 and 1000 mV for all temperatures tested.

**Figure 4:**
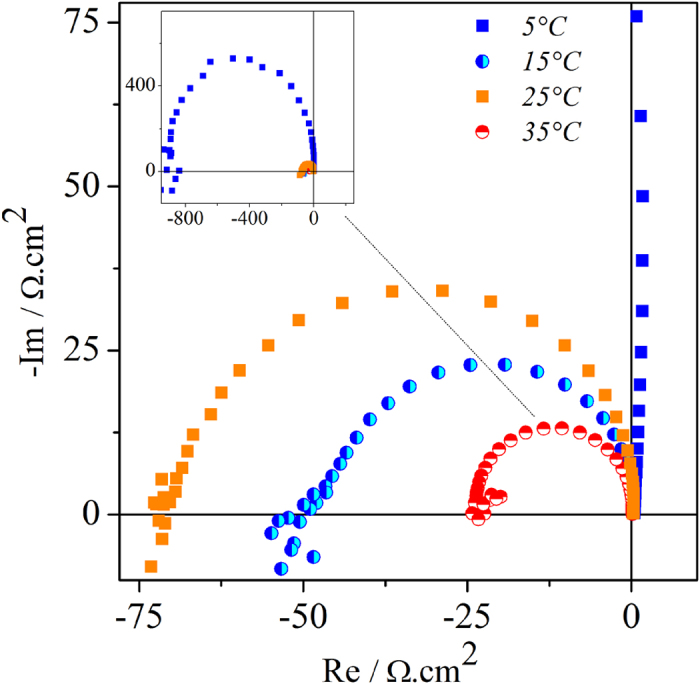
Impedance spectra registered at 800 mV for different temperatures. Full scale is shown in the inset.

Nyquist plots showing the general tendency of the temperature dependence on the semi-circles obtained are depicted in Fig. 4. The single semi-circles become smaller with increasing temperature, but there is also seen a break of this tendency for the experiments at 25 °C. Important in the present analysis, EIS can also be used qualitatively to identify Hopf and saddle-node bifurcations in an electrochemical system. Ultimately, a bifurcation is a situation where the impedance spectrum intersects or ends up in the origin of the complex impedance plane. If it cross the negative side of the real axis as *ω* → 0 it corresponds to a saddle-node bifurcation; if however and if the spectrum intersects the negative side of the real axis at a non-zero frequency, the system undergoes a Hopf bifurcation^4^. In this sense, Hopf bifurcation frequencies *ω*_*Hopf*_ were obtained by EIS at different DC potentials, at different temperatures for the formic acid system. We found increasing values of Hopf frequency up to 25 °C, with subsequent decrease for T > 25 °C, corroborating with the tendency obtained under galvanostatic conditions.

**Figure 5:**
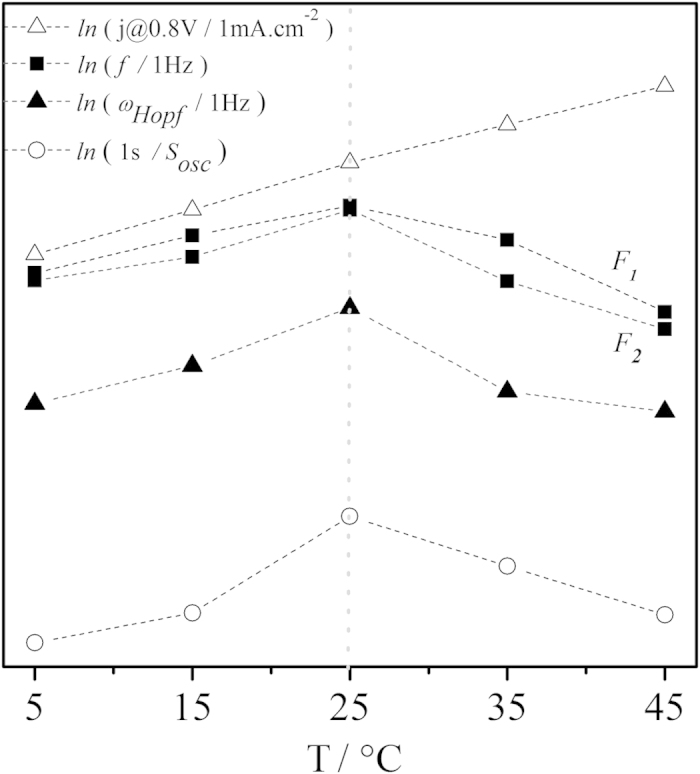
Temperature effect on frequencies *f* (full squares) *ω*_*Hopf*_ (full triangles) and *S*_*osc*_(empty circles) showing a similar break of tendency at 25 °C. Hopf bifurcation frequencies obtained at 700 mV. For comparison, the voltammetric current at 800 mV obtained along the positive going sweep, c.f. Fig. 1a, is also presented.

Figure 5 summarizes the temperature dependence on most experimental parameters discussed so far. In contrast to the regular dependence observed for the voltammetric current, i.e. higher rates at higher temperatures, the behavior found in all variables studied under oscillatory regime shows a turning point at 25 °C. Activation energies estimated in the range between 5 and 25 °C vary roughly from 40 to 50 kJ mol^−1^, and, between 25 and 45 °C, from −70 to −95 kJ mol^−1^. Interestingly, comparable behavior was found in the three oscillation frequencies studied. Implications of these results are discussed in the next section.

## Discussion

We report the experimental study of the effect of temperature on the fast and slow dynamics observed in a model oscillatory surface reaction, namely the electro-oxidation of formic acid on polycrystalline platinum and in acidic media. Following our previous investigations^7^, we explored the nonlinearities intrinsic to this reaction in order to deepener our understanding on the coupling between the two disparate time-scales responsible for the oscillations themselves and for the long-term surface deactivation. The main aspect to be discussed in this seminal approach is the unexpected existence of a turning point in the dynamics at 25 °C, at which the temperature dependence of most variables changes. Now we discuss important dynamic and mechanistic information that result from these empirical observations.

**Figure 6:**
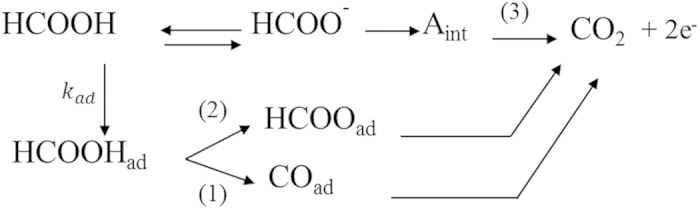
Schematic representation of the multiple parallel routes for the oxidation of formic acid on platinum, in acidic media. Stoichiometry is neglected for the sake of simplicity.

The electro-oxidation of formic acid on platinum and on platinum-based surfaces has been extensively studied as a model surface reaction, with application in energy conversion devices such as low temperature fuel cells^31^. Despite its structural simplicity, formic acid is oxidized in a complex triple parallel pathway, which can be reasonably represented by the scheme given in Fig. 6.

The reaction scheme involves the adsorption of HCOOH followed by a non-faradaic dehydration of HCOOH_ad_ (1) and the formation of chemisorbed carbon monoxide, CO_ad_. Formic acid also proceeds to adsorbed formate (2) via two oxygen atoms, occupying two surface sites^32^. Due its high stability, formate is unlikely to be a reactive intermediate to CO_2_ and might be in equilibrium with its soluble form acting as a mere blocking spectator at lower voltage^33^, in contrast with the case of methanol^34^. In practical terms, it is safe to assume that formic acid is mainly oxidized through a non-formate pathway. For the main route, the direct pathway is believed to possess the lower activation energy^35^ and the formation of carbon dioxide occurs via a weakly adsorbed intermediate, step (3), formed after the activation of the C-H bond^36^, whose nature is still matter of debate. Feliu and co-workers^33^ have recently published some new insights on the mechanism of formic acid system in which the authors suggest formate anion interacting with the surface in a C-H down configuration as the active intermediate, *A*_*int*_. At considerably high potentials, the formation of adsorbed oxygenated species occurs, as generically represented by the step (4):


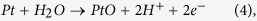


and it can assist the oxidation of chemisorbed carbon monoxide via a Langmuir-Hinshelwood (LH) step (5):


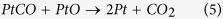


The effect exerted by the temperature in an electrocatalytic reaction such as the electro-oxidation of small organic molecules discussed here depends on parameters including the surface structure, the applied potential, the dominant current carrier step at the state being investigated, the relative surface coverage of adsorbates, among others^23^. Therefore, even under non-oscillatory conditions, the analysis of the apparent activation energy is a non-trivial task. Results presented in Fig. 1 reveal conventional temperature dependence under voltammetric regime. Simple Arrhenius dependence was found in this extended interval studied, and representative apparent activation energies found in the range between 40 and 60 kJ mol^−1^ are in agreement with previously published data^23^,^25^,^33^.

During oscillations, the temperature dependence is even more intricate as more than one elementary step is probed in the potential window visited. The potential oscillations obtained at constant applied current reflect the continuous changes in the population of the different adsorbed species, such as CO_ad_, HCOO_ad_, and oxygenated species;^37^ transport of species to/from the electrode surface is unlikely to be responsible for the observed qualitative differences^38^. Comprehensive description and mechanism of the oscillatory electro-oxidation can be found elsewhere^28^.

The temperature dependence of steps (1)–(5) globally controls the response under both voltammetric and galvanostatic regimes in different ways. In the first case, the distribution of apparent activation energies, c.f. Fig. 1, reflects the temperature dependence of the rate-determining step at a given applied potential. Under oscillatory regime, different steps are simultaneously probed and the activation energy estimated by global quantities, such as the oscillatory frequency, is a combination of different steps. In this respect, we studied the effect of temperature in terms of the oscillatory frequency for two families of oscillations^7^. In order to infer on the intrinsic dynamics near the Hopf bifurcation we also estimated the Hopf frequency via impedance spectroscopy, c.f. Fig. 4.

As showed in Fig. 5, conventional Arrhenius behavior prevails for temperatures up to 25 °C when the subsequent increase of temperature reveals an opposite, counter-intuitive, tendency: the oscillation frequency decreases for a temperature increase. The latter behavior is referred to as temperature overcompensation and corresponds to a situation of negative apparent activation energy. Temperature (over)compensation has been found in the oscillatory electro-oxidation of formic acid^7^,^39^,^40^, and ethylene glycol^54^. Important to the present context, in all these cases, this unusual behavior was proved to emerge from the intricate combination of several reaction steps, *vide infra*.

As for the long-term evolution, it has been shown that even though all experimental parameters are carefully controlled, the system slowly evolves and visits distinct dynamic states, c.f. oscillations of type F_1_ and F_2_, up to the end of oscillations. The slow spontaneous changes that drive the system occurs in a much slower time-scale than that found for the oscillations themselves, referred here to as core oscillator. The time-scales separation (*τ*), estimated as the ratio between the total oscillation time and typical oscillation period, is about three orders of magnitude, c.f. Supplementary Table S1. The impact exerted by temperature on the long-term evolution was studied by means of the size of the oscillatory region, *S*_*osc*_, obtained for a given normalized applied current, Fig. 2. As seen in this figure, *S*_*osc*_ initially decreases with temperature from 5 to 25 °C (note that 1/*S*_*osc*_ is plotted in Fig. 5), and then it increases in the higher temperature domain. This behavior would apparently imply that the rate of the slow drift is first accelerated with the temperature increase and then slowed down for 25 °C < T ≤ 45 °C. This is however an oversimplified description as the slow drift is chemically coupled to the core oscillator. Before a more appropriate reasoning, we present in the following the current understanding of the mechanism behind this slow changes.

The slow drift observed in oscillatory electrochemical time-series have been long observed and generally attributed to surface processes rather than bulk phenomena such as mass transport or homogeneous reactions^41^,^42^. We have studied in depth this phenomena in the electro-oxidation of small organic molecules on platinum in acidic media and proposed a mechanism^43^,^44^. In short, when the formation of surface oxygenated species reaches a certain limit, the place exchange phenomena^45^,^46^ results in the insertion of these species into the platinum lattice, which can be represented generically as,


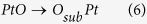


Consequently, the subsurface oxygen, *O*_*sub*_, becomes unavailable to assist the surface oxidation of adsorbed carbonaceous species such as *PtCO*, and also the site *O*_*sub*_*Pt* becomes less active to catalyze most steps. The accumulation of subsurface oxygen occurs in a much slower time-scale than the potential oscillations and it can be inferred by the increase of the mean potential^43^ and also by the accumulation of *PtCO*, in the case of methanol^44^.

**Figure 7:**
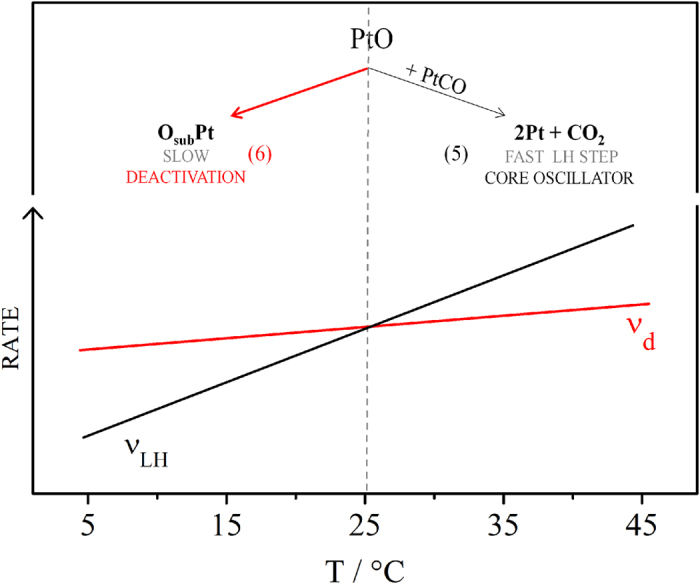
Schematic representation of the coupled slow and fast dynamics in the formic acid galvanostatic oxidation and the temperature dependence observed.

Bringing together the current understanding of the mechanism of the core oscillator and of the slow surface deactivation, it becomes evident the importance of *PtO* species: it can either assist the oxidation of, say, adsorbed carbon monoxide, step (5), or form *O*_*sub*_*Pt* and deactivate the surface, step (6). Therefore, *PtO* species chemically couple slow and fast dynamics. The results obtained for the effect of temperature on the size of the oscillatory window, *S*_*osc*_, can be now discussed in terms of Fig. 7, which schematically illustrates the rates of the Langmuir-Hinshelwood (LH) step (5), ***ν***_LH_, and of the slow deactivation process (6), ***ν***_*d*_.

As the main idea introduced in this work, temperature was used to infer on the relative rates of the processes involved in the disparate and coupled time-scales intrinsic to the system. Figure 7 illustrates the two temperature domains observed that can be explained in terms of the *PtO* species. In the low temperature domain, the decrease of the oscillatory window with temperature evidences that the rate ***ν***_*d*_ is higher than that of the LH step. In the same way, from 25 to 45 °C the LH step is faster and the consumption of oxygenated species *PtO* in the oxidation of adsorbed carbon monoxide, step (5), prevents the place exchange phenomena, step (6), and the time-series becomes longer with the temperature increase, c.f. Fig. 2. Focusing now on the whole temperature range illustrated in Fig. 7, it becomes clear that the activation energy of the LH step is higher than that of the slow surface deactivation, previously attributed to the place exchange phenomena. This observation can also be related to data available in the literature, obtained under non-oscillatory conditions. In fact, activation energies of at least about 100 kJ.mol^−1^ have been found for the electro-oxidation of carbon monoxide on platinum single crystals^47^. In contrast, it seems there is a rather very weak dependence on temperature in the oxide formation step, and presumably on the place exchange process^48^. It has been shown^49^,^50^ that, for a temperature range of 278 ≤ T ≤ 323 K, the increase of oxide formation and reduction is about 26%, under comparable conditions. Moreover, the activation energy for the direct oxidation of formic acid was found to vary from 50 to 60 kJ.mol^−1^ and its dependence on the electrode potential rationalized in terms of the changes in the coverage of anions and oxygenated species^22^,^51^.

An interesting aspect presented in our results is the turning point found for many variables at 25 °C. The formation of adsorbed CO on platinum from formic acid is essentially a chemical step, which has, however, an electrochemical nature since the dehydration of formic acid is potential-dependent. Data available in the literature, obtained by means of CO_ad_ stripping add more important information to the matter, see ref. 47 and references therein. The onset potential for CO_ad_ oxidation significantly shifts towards less positive values as temperature is increased and, in addition to the oxidation charge, the authors indicate a change in the stability of the CO_ad_ adlayer depending on the temperature used. A very interesting result is that the CO_ad_ oxidation charge for Pt(111) shows decreasing values from 270 to 298 K (having its minimum value at 298 K) with a slight increase in the range 298 K < T < 323 K. At 298 K the authors observed the lowest CO coverage(*θ*_*CO*_). Once the oscillations found here shows shortest stability window at this very temperature, it seems reasonable to infer that higher oscillatory frequencies are observed for smaller maximum critical *θ*_*CO*_. Taking into account that the time necessary to reach a certain critical *θ*_*CO*_ must increase as *θ*_*CO*_ increases in oscillatory conditions, the observance of *f* increasing for 5° < T < 25° and decreasing for 25° < T < 45 °C can be related to the experimental findings^47^ for the temperature dependence on *θ*_*CO*_. In addition, EIS data obtained in the present work reveal smaller values for double layer capacitance, C_dl_, at 25 °C in comparison with the neighbour values of temperatures tested, strongly suggesting a less blocked surface at this temperature, as shown in Supplementary Fig. S3. Despite the experimental differences (mainly surface structure, species being oxidized, and the reaction regime itself), it is remarkable the turning point found at 25 °C. The way in which the increase in the CO coverage above 25 °C^47^ and the higher rates of the LH step observed here during oscillations regime are under numerical investigation at the moment in our Group.

Back to the temperature dependence of the core oscillator, unusual behavior such as temperature compensation and overcompensation can occur in a network of chemical reactions and be reflected in responses such as the constancy of the oscillatory frequency. Overall, these unconventional temperature dependencies can be conveniently rationalized with the concept of antagonistic balance in which period increasing and decreasing sets of elementary reactions and their respective activation energies are combined to result in oscillatory apparent activation energy^52^,^53^. As the region in which the oscillation frequency decreases with temperature, i.e. T > 25 °C, occurs where the LH step, (5), is dominant, we can speculate that this step belongs to the *period-increasing* set. It is important to recall that there is no available method to classify the individual steps as it is difficult to isolate a given elementary reaction and estimate its role when part of a network.

## Conclusions

We report the effect of temperature on the disparate time-scales observed in a model oscillatory surface reaction, namely the electro-oxidation of formic acid on polycrystalline platinum and in acidic media. Already in the short-term dynamics (or of the core oscillator), we found a turning point at 25 °C, where the temperature dependence of most variables changes noticeably. This turning point was also found in the parameter used to study the long-term dynamics. The two regions are characterized by a conventional Arrhenius behavior between 5 °C and 25 °C, and by non-Arrhenius dependence for temperatures between 25 °C and 45 °C, where essentially, the oscillation frequency decreases as the temperature increases. This behavior was related to previously published data on the turning point on the coverage of adsorbed carbon monoxide at 25 °C and also corroborated by the impedance data presented.

The current understanding of the dynamics of the core oscillator and of the slow surface deactivation stresses the dual and pivotal role of *PtO* species, as it assists the oxidation of adsorbed carbon monoxide and forms *O*_*sub*_*Pt*, the long-term surface-blocking species. The competition between these two processes was explained in terms of long-term evolution and the turning point at 25 °C. Based on these findings, we were able to compare the activation energies of both steps and clearly attribute a weaker temperature dependence of the slowly evolving surface deactivation. This observation is also corroborated by independent experimental results and, importantly, obtained under non-oscillatory conditions. The described competition between the two steps also allowed us to speculate on the role of the surface oxidation of carbon monoxide by *PtO* species as being part of the set of reactions that contribute to the increase of the oscillation period with temperature.

The study of temperature effect on both short and long-term dynamics introduced here can be though as a methodology to deepen the current knowledge on some mechanistic aspects. The discrimination of the elementary reactions belonging to the period-in/decreasing sets and the also the determination of activation parameters for individual steps are particularly important to uncover the present and also more complex mechanisms. We are currently exploring such aspects using similar systems and also other experimental parameters, such as the influence of different surface-blocking species.

## Methods

A conventional glass cell equipped with three electrodes was used in our experiments. As working electrode we used smooth polycrystalline platinum with a geometric area of 0.2 cm^2^ and roughness factor between 2.0 and 2.5. Current densities (j) were calculated dividing the current applied by the electrochemical active area, which in turn was calculated using the well accepted charge for UPD hydrogen region as 210 μC.cm^−2 ^11,54. A reversible hydrogen electrode (RHE) was prepared using the same solution employed as supporting electrolyte and was chosen as reference electrode. All potentials were corrected and are referred against RHE at 298 K^55^. A large-area platinum electrode was used as counter electrode. All solutions were prepared using ultra-pure water (Milli-Q system, Millipore, 18.2 MΩ.cm), sulfuric acid (Merck, 98% purity) and formic acid (Sigma-Aldrich, purity ≥98%). The electrochemical measurements were all done by means of a potentiostat/galvanostat PGSTAT 30 (Autolab) equipped with SCANGEN and FRA modules. The temperature of the bulk solution was varied and maintained through a thermostatic bath, monitored using a K-type thermocouple (precision of ± 0.5 K). Electrochemical impedance spectroscopy (EIS) was performed in the frequency range from 10 kHz to 0.01 Hz, applying an AC modulation of 10 mV. To assure stationary state, chronoamperometric transients at the identical DC potential to be applied were recorded for at least 500 s after a time-invariant profile had been reached (dj/dt = 0). All experiments described here were performed at [HCOOH] = 1 M dissolved in aqueous [H_2_SO_4_] = 0.5 M.

## Additional Information

**How to cite this article**: Zülke, A. A. and Varela, H. The effect of temperature on the coupled slow and fast dynamics of an electrochemical oscillator. *Sci. Rep.* **6**, 24553; doi: 10.1038/srep24553 (2016).

## Supplementary Material

Supplementary Information
