# Supplementary material for: The effect of temperature on the coupled slow and fast dynamics of an electrochemical oscillator
Source: Sci Rep. 2016 Apr 15;6:24553. doi: 10.1038/srep24553 (PMC4832193; doi:10.1038/srep24553)
Supplement: Supplementary Information [file srep24553-s1.pdf]

The effect of temperature on the coupled slow and fast dynamics of an electrochemical oscillator

Alana A. Zülke and Hamilton Varela*

Institute of Chemistry of São Carlos, University of São Paulo

POBox 780, 13560-970, São Carlos, SP, Brazil

Supplementary Information

The main qualitative and quantitative information extracted from the potential time series ($J_{0.5}^{T}$) are summarized in Table S I **:**

SUPPLEMENTARY TABLE S1:

TABLE SI Description of the average features observed for the oscillatory dynamics at different temperatures

T (°C)	$\bar{f}$ */ Hz**		*%S_{osc}***		$\overline{\Delta E}$ */ mV**		*Waveform*		τ ***	
	F_1	F_2	F_1	F_2	F_1	F_2	F_1	F_2	F_1	F_2
5	0.5	0.8	23.8	19.0	240	110	P^1	P^1	936	1400
15	0.9	1.5	18.0	18.8	250	115	P^1	P^1	900	1500
25	2.5	3.0	28.0	43.7	290	200	P^1,P^2	P^1 , P^2	500	750
35	0.6	1.6	24.3	36.5	280	190	P^1,P^2	P^1 , P^2	250	664
45	0.2	0.4	18.2	9.4	275	150	P^1,P^2	P^1 , P^2	200	400

Average value for at least 5 cycles. **Average percentage from the overall S_{osc} spent by each family of oscillations with respect to the whole window.*Estimation of time-scale separation as average value of at least 5 cycles ($\tau = \overline{S_{osc}} \times \bar{f}$).*

Three oscillatory families common to all temperature tested were classified according to potential amplitude, frequency and waveform as shown for a typical response presented in Fig. S1:

SUPPLEMENTARY FIGURE S1:

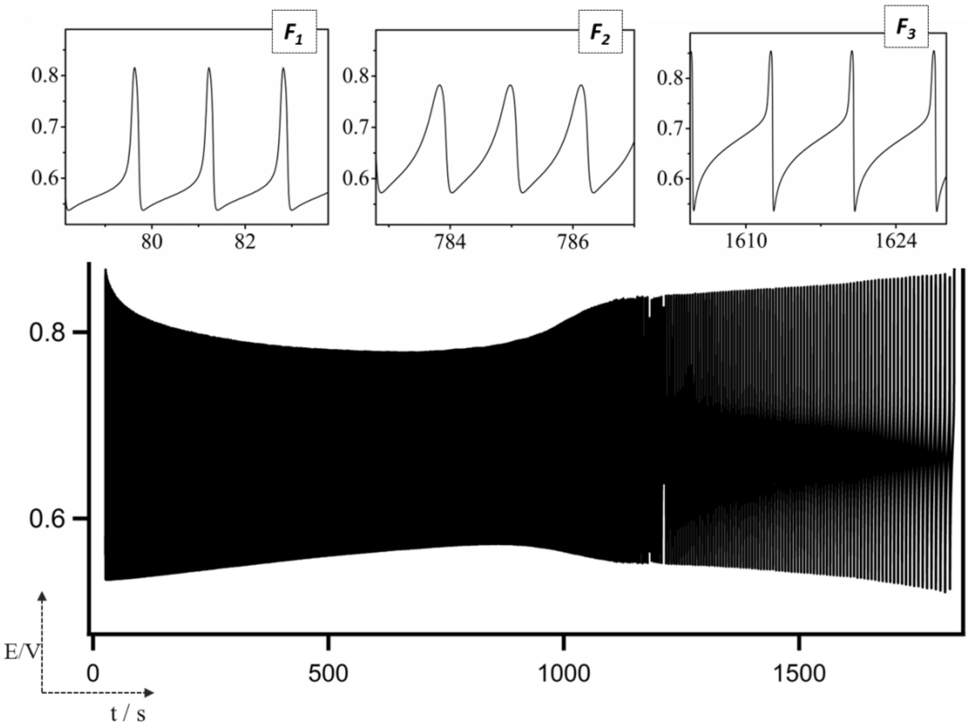

Figure S1 On the top: Three zoomed regions of the potential time series (complete time series shown on the bottom) displaying three distinguishable oscillatory families. Data measured for the system at $J_{0.5}^{5°C}$.

The distributions of frequency for the time series ($J_{0.5}^{T}$) can also be compared by evaluating the FFT spectra, displayed as magnitude versus frequency (Hz) :

SUPPLEMENTARY FIGURE S2:

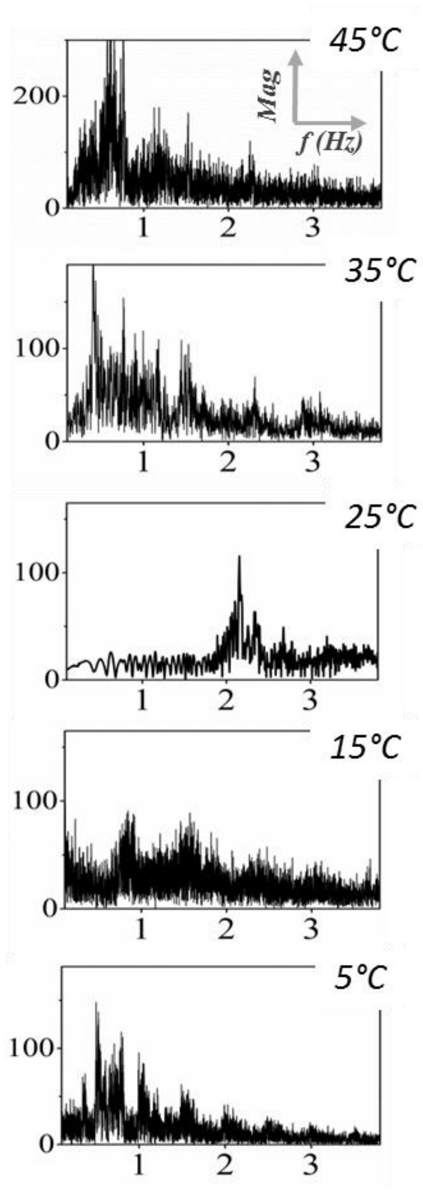

Figure S2: FFT Spectra $J_{0.5}^{T}$ at different temperature as indicated. Magnitude versus frequency (Hz);

Activation parameters for the oscillatory dynamics were then estimated assuming the formalism for Arrhenius-type dependency of the temperature of f and S_{osc} obtained by galvanostatic measurements maintaining the same normalized current applied j , and ω_{hopf}, obtained by EIS at the same DC potential, E. For all the latter variables used to estimate activation parameters, the same dual behavior was found: a negative slope from 5-25°C revealing positive values of apparent activation energy and a region above 25°C displaying the counter intuitive concept of negative apparent activation energy. Values for activation parameters were then extracted for the Arrhenius-type of behavior shown Fig. 5, by (i), (ii) and (iii) as 44$\pm$1 $kJmol^{-1}$ (for F_1) and 46$\pm$2$kJmol^{-1}$ (for F_2); 65$\pm$17$kJmol^{-1}$, 62$\pm$3$kJmol^{-1}$, respectively.

$$\text{(i)} - R\left[\frac{\partial\ \ln(\mathrm{f}/1\mathrm{Hz})}{\partial \mathrm{T}^{-1}}\right]_{J_n^T, F_i} \qquad \text{(ii)} - R\left[\frac{\partial\ \ln(\ 1\mathrm{s}/\mathrm{S}_{osc})}{\partial \mathrm{T}^{-1}}\right]_{J_n^T} \qquad \text{(iii)} - R\left[\frac{\partial\ \ln\left(\omega_{\mathrm{Hopf}}/1\mathrm{Hz}\right)}{\partial \mathrm{T}^{-1}}\right]_{E}$$

Double layer capacitances (C_{dl}) extracted from the impedance data were analyzed and some typical results for the temperature dependence are shown in Fig. S3:

SUPPLEMENTARY FIGURE S3:

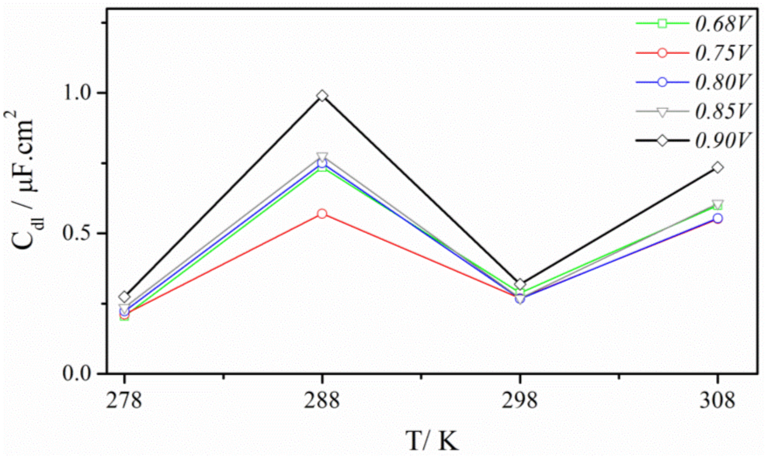

Figure S3: Temperature dependence on the double layer capacitance at different DC potentials as indicated.
